# Chronic Lymphocytic Inflammation With Pontine Perivascular Enhancement Responsive to Steroids: An Acute Presentation

**DOI:** 10.7759/cureus.21382

**Published:** 2022-01-18

**Authors:** Ayesha R Ambia, Norah AlZahrani, Abdul Hakim Almakadma, Tasnim A Elgazzar, Sami Almustanyir

**Affiliations:** 1 College of Medicine, Alfaisal University, Riyadh, SAU; 2 Neurology, Prince Mohammed Bin Abdulaziz Hospital, RIyadh, SAU; 3 Internal Medicine, Minsitry of Health, Riyadh, SAU

**Keywords:** gadolinium enhancement mri, clippers syndrome, corticosteroid therapy, case report, gait ataxia

## Abstract

Chronic lymphocytic inflammation with pontine perivascular enhancement responsive to steroids (CLIPPERS) is a rare disease with an unknown etiology which most commonly results in subacute diplopia and ataxia. Diagnosis is achieved through a triad of the following findings: lymphocytic pleocytosis with increased CD4+ T cells on cerebrospinal fluid (CSF) analysis; perivascular punctate and curvilinear hemorrhages in the pons, medulla, or cerebellum on magnetic resonance imaging (MRI) with contrast; and the cessation of symptoms after the initiation of corticosteroids. Here, we report the case of a 23-year-old male who presented with non-specific signs and symptoms, including diffuse weakness in all limbs, ataxia, and slurred speech. The diagnosis was achieved through a contrast MRI of the brain, suggestive of brainstem encephalitis, and a CSF analysis, which revealed elevated glucose and protein levels. Intravenous methylprednisolone was administered for five days and resulted in acute improvement of the patient’s clinical status. Repeat CSF analysis and MRI of the brain with contrast two weeks later showed resolution of previous findings. CLIPPERS syndrome is a newly identified disease thought to cause a predominantly inflammatory reaction in the pons, medulla, cerebellum, and supratentorial region. MRI with contrast tends to reveal a “salt and pepper appearance” in a punctate and curvilinear fashion. The hallmark of treatment is corticosteroid therapy, and discontinuation of therapy should be done with caution as relapse of the syndrome with corticosteroid withdrawal has been documented.

## Introduction

Chronic lymphocytic inflammation with pontine perivascular enhancement responsive to steroids (CLIPPERS) syndrome is a rare disease of unknown etiology, with approximately 60 cases described worldwide. It predominantly affects men over the age of 50 years and presents with a wide range of symptoms such as diplopia, ataxia, or spasticity. The cerebrospinal fluid (CSF) analysis usually reveals high protein and lymphocytic pleocytosis, with CD4+ predominant cells. The diagnosis of CLIPPERS is achieved through a triad of clinical and radiological findings, including, magnetic resonance imaging of the brain (T2-weighted fluid-attenuated inversion recovery [FLAIR] sequences revealing “salt and pepper” sign) revealing heterogeneous hyperintensities less than 3 mm in size [1], CSF analysis showing lymphocytic pleocytosis, and clinical improvement with corticosteroid administration. CLIPPERS is an inflammatory neurological disease affecting mainly the pons and cerebellum and less commonly the spinal cord. Other regions include the cerebral peduncles, medulla, and midbrain. A perivascular hyperintense pattern is common in pontine and peripontine areas. The hallmark of diagnosis and treatment for CLIPPERS is the use of immunosuppressive glucocorticoids, which immediately relieve symptoms. Corticosteroids are required for long-term management to prevent relapse [2].

## Case presentation

A 23-year-old male with type 1 diabetes presented to the Emergency Department (ED) with confusion, slurred speech, and diffuse limb weakness (3+ throughout) for two days. His medical history was otherwise unremarkable. The patient was afebrile and his vitals were within normal limits. Blood glucose levels were 40 mg/dL and were immediately corrected with no change in his clinical condition. Complete blood count and comprehensive metabolic panel revealed no abnormalities. Erythrocyte sedimentation rate was 74 mm/hour and HbA1c was 13.5%. Initial computed tomography (CT) of the head without contrast was grossly unremarkable and revealed no intracranial hemorrhage or acute territorial infarction. Subsequently, a CT venogram was performed which ruled out cerebral sinus thrombosis. An ultrasound carotid Doppler showed no stenotic or atheromatous plaques, patent vessels, and normal flow. A cardiac echocardiogram showed normal hemodynamic capacity with an ejection fraction of ≥55%.

**Figure 1:**
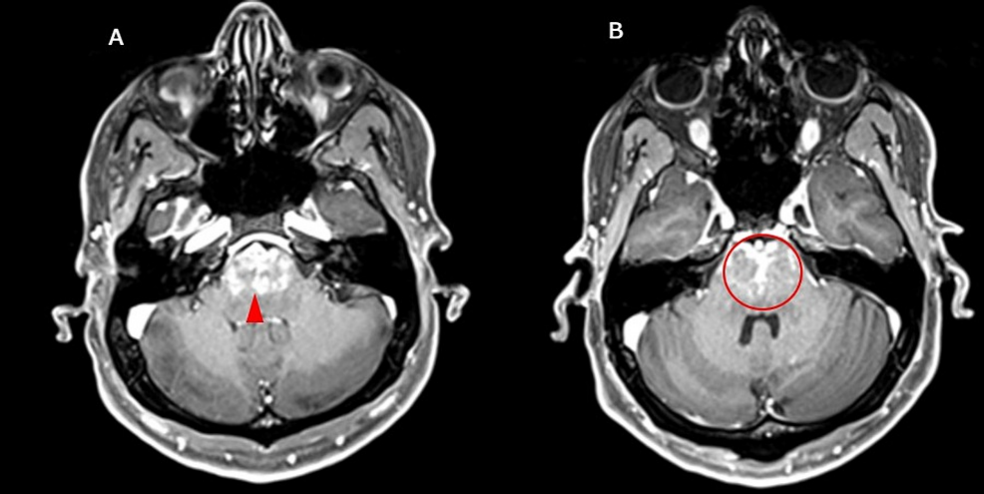
Axial brain T1-weighted MRI with contrast showing (A) hyperintense signal (arrowhead) involving the pons and (B) heterogeneous enhancement (encircled) in the pons on the post-contrast study. MRI: magnetic resonance imaging

**Figure 2:**
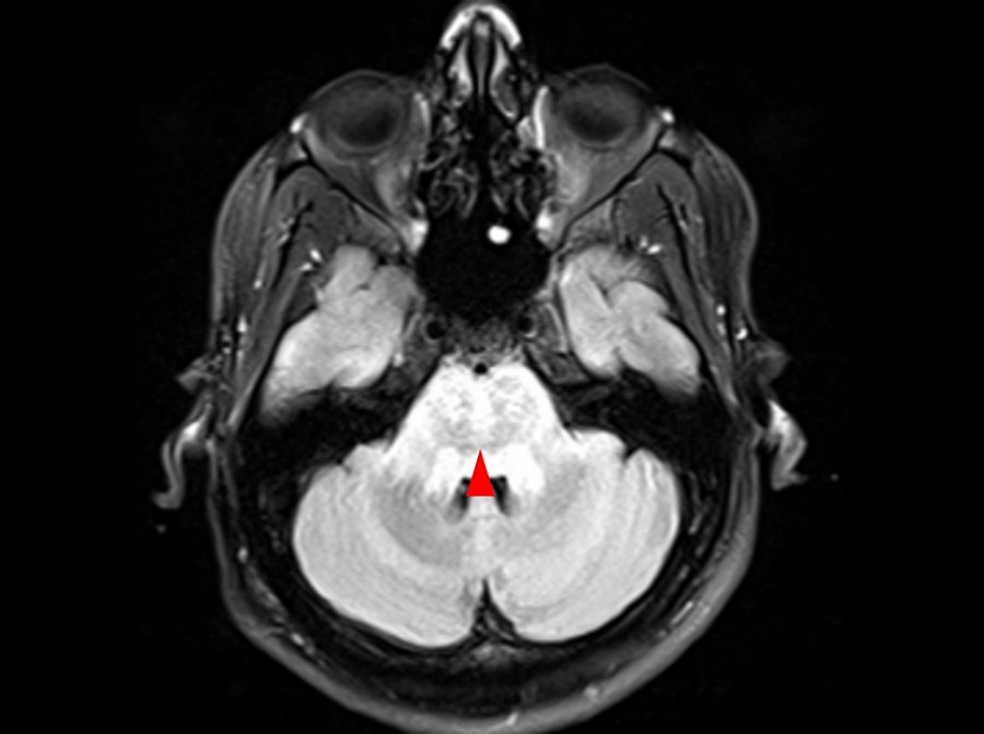
Axial MRI FLAIR sequence showing a hyperintense signal mainly involving the pons (arrowhead). MRI: magnetic resonance imaging; FLAIR: fluid-attenuated inversion recovery

Hence, magnetic resonance imaging (MRI) with contrast was deemed necessary. On MRI, there was moderately extensive pontine edema with a few foci of diffusion restriction in the contrast study (Figure 1A) and heterogeneous enhancement on the post-contrast study (Figure 1B) extending into the midbrain, medulla oblongata, and bilateral cerebral peduncles. The FLAIR signal was also hyperintense (Figure 2). The cranial nerve examination was normal; there were no lesions in the cerebellum nor was there any indication of a cerebellar herniation. These non-specific findings could manifest because of infectious etiologies such as brainstem encephalitis, non-infectious causes such as osmotic demyelination syndrome, or an isolated brainstem reversible hypertensive encephalopathy. The post-contrast MRI was suggestive of brainstem encephalitis, for which CSF analysis was performed. CSF analysis revealed a glucose level of 9.9 mmol/L, a protein level of 403 mg/dL, and 9 leukocytes/μL with negative gram stain and culture. Magnetic resonance angiography (MRA) of the brain showed normal patent vessels, and vasculitis screening was negative.

**Figure 3:**
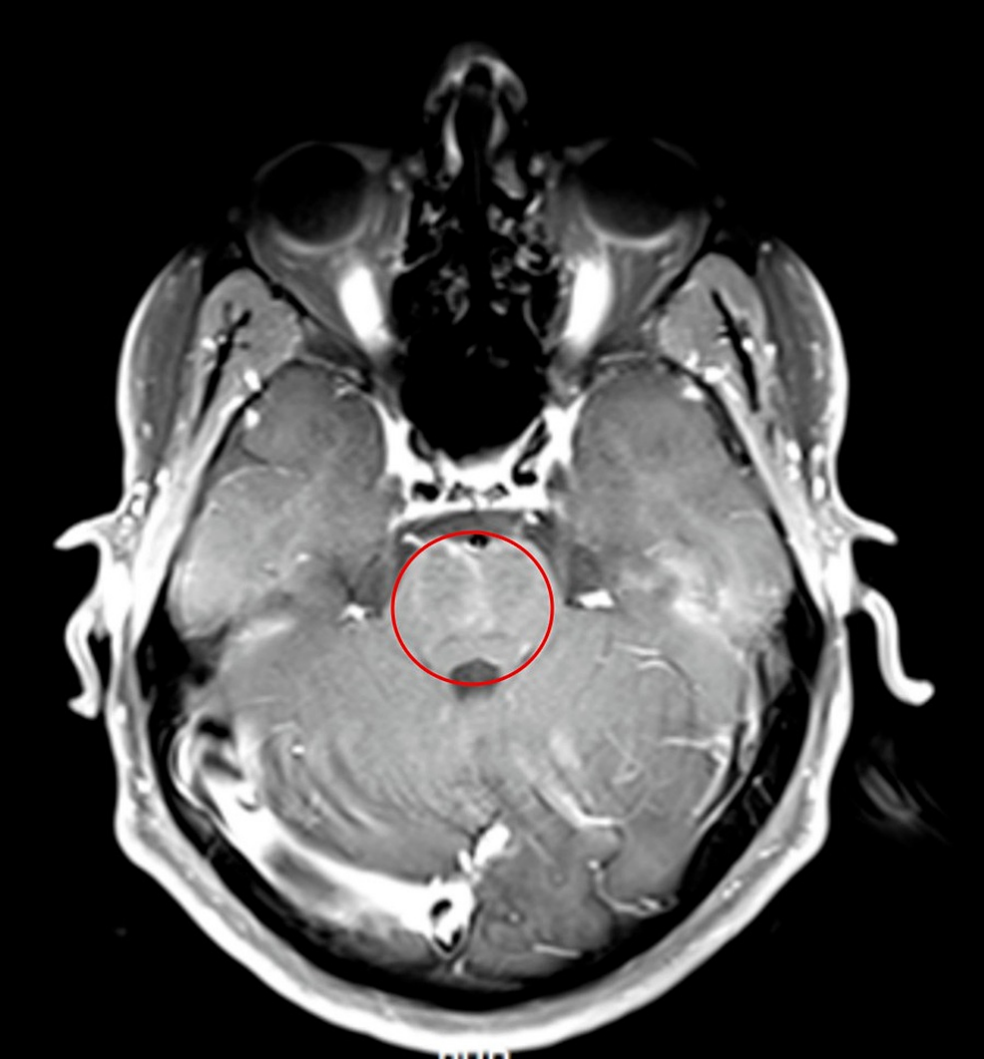
Axial MRI T1-weighted image with contrast sequence obtained post-treatment with pulse steroid showing signal improvement involving the pons (encircled). MRI: magnetic resonance imaging

**Figure 4:**
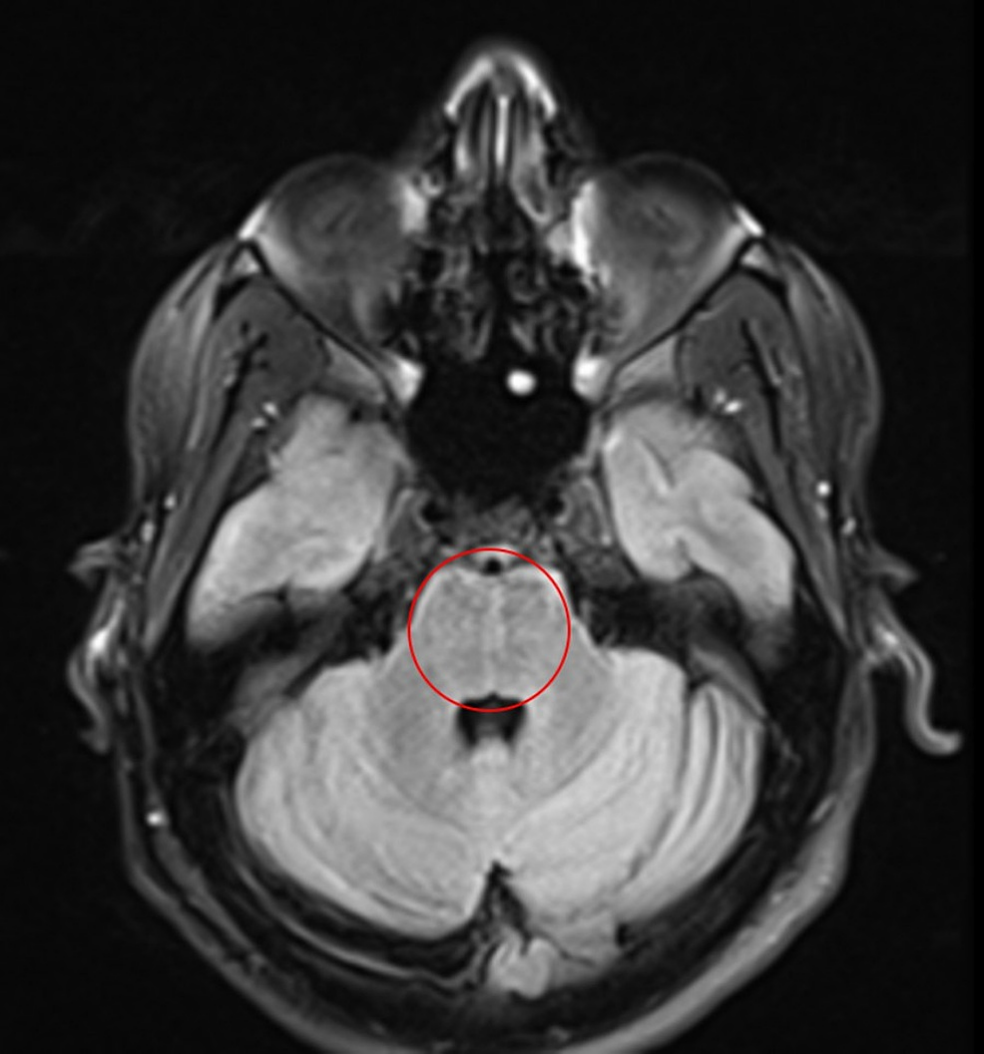
Axial MRI with FLAIR sequence showing signal resolution in the pons after administering pulse steroids (encircled). MRI: magnetic resonance imaging; FLAIR: fluid-attenuated inversion recovery

After administering 1 g of intravenous (IV) methylprednisolone for five days, acute improvement in the patient’s clinical status was observed. Repeat investigations were performed. MRI with contrast showed further interval treatment with substantial resolution of signal change and abnormal enhancement of the pons (Figure 3). The only salient finding was that of a slight hyperintense signal in the central pons and bilateral curvilinear enhancement in the anterior pons (Figure 3). A signal resolution was also achieved in the FLAIR sequence (Figure 4). The clinical presentation, CSF analysis, and MRI findings combined with the rapid response to corticosteroid therapy with significant clinical improvement made CLIPPERS the most likely diagnosis. Rapid improvement of symptoms after initiation of steroids has also been documented previously in other cases of CLIPPERS [3]. The patient was discharged on insulin therapy and oral prednisone for a month. He was scheduled for his follow-up visit as well as a follow-up MRI in two months.

## Discussion

CLIPPERS syndrome was first described in 2010 by Pittock et al. [4]. It often presents with a triad of neurological signs and symptoms, a dense perivascular CD4+ T cell infiltrate, and clinical improvement after initiating glucocorticoid therapy [5]. The brainstem findings seen in CLIPPERS include punctate or “salt and pepper” perivascular enhancement of the MRI in the pons, cerebellum, or brachium pontis [6,7]. Some less known symptoms of CLIPPERS syndrome include nystagmus, tinnitus, tremor, nausea, paraparesis, spasticity, allodynia, asthenia, and cognitive impairment.

Although the disease presents in a relapsing and remitting fashion, sufficient features of systemic and autoimmune diseases have still not been met by CLIPPERS. Consequently, multiple investigations are often performed to rule out systematic and autoimmune diseases [8,9]. Furthermore, post-inflammatory axonal injury and development of lymphoma after CLIPPERS have been discussed in the literature [10], with very little understanding of the underlying pathophysiology of the disease. CLIPPERS syndrome shows similar MRI characteristics typical in various disorders, such as primary central nervous system angiitis, lymphoma, or multiple sclerosis. MRI findings in these patients show a pathognomonic perivascular T cell infiltration of the pons, which can extend to the supratentorial region [11].

Our patient’s non-specific clinical and radiological findings suggested multiple possible differentials such as encephalitis, osmotic demyelination syndrome, or isolated brainstem reversible hypertensive encephalopathy [2]. Our patient fulfilled the MRI diagnostic criteria of CLIPPERS with heterogeneous punctate lesions and swift improvement in clinical status with corticosteroid initiation. As such, the diagnosis of CLIPPERS syndrome was made. The mainstay of treatment is through the use of corticosteroid therapy. However, withdrawal of steroid therapy has been shown to result in disease relapse [12]. Management of relapse of CLIPPERS includes additional therapy of methotrexate, cyclophosphamide, azathioprine, or tocilizumab [7,8,13]. A multi-center study performed from 1999 to 2009 included eight patients with CLIPPERS, followed up for a median of 22 months (7-144 months). All patients showed characteristic curvilinear enhancement on gadolinium enhancement which worsened on discontinuation of corticosteroid therapy [4]. The decision of tapering steroids should be made with caution and possible corticosteroid-sparing therapies should be used as an alternative for long-term management.

## Conclusions

Patients with CLIPPERS syndrome generally present with non-specific neurological symptoms in a subacute fashion that suggest lesions in the brainstem region. With non-specific focal neurological signs, it can be diagnosed initially as a myriad of other neurological diseases, which can cause added discomfort to the patient. Therefore, we recommend keeping CLIPPERS syndrome as a diagnosis of exclusion, especially when no other definitive diagnosis can be reached. Apart from clinical diagnosis, we recommend the use of MRI and CSF analysis as initial investigations when no other etiology is found. The search for an answer to CLIPPERS syndrome possibly being an autoimmune or a systemic disease due to its relapsing and remitting features can be an important milestone in learning more about this disease and its pathogenesis.
